# Initial Development and Psychometric Evidence of Physical Education Grit Scale (PE-Grit)

**DOI:** 10.3389/fpubh.2022.818749

**Published:** 2022-03-03

**Authors:** Noomen Guelmami, Nasr Chalghaf, Amayra Tannoubi, Luca Puce, Fairouz Azaiez, Nicola Luigi Bragazzi

**Affiliations:** ^1^Postgraduate School of Public Health, Department of Health Sciences (DISSAL), University of Genoa, Genoa, Italy; ^2^Group for the Study of Development and Social Environment (GEDES), Faculty of Human and Social Science of Tunis, Tunis, Tunisia; ^3^Department of Human and Social Sciences, Higher Institute of Sport and Physical Education of Kef, University of Jendouba, Jendouba, Tunisia; ^4^Department of Human Sciences, Higher Institute of Sport and Physical Education of Sfax, University of Sfax, Sfax, Tunisia; ^5^Department of Neuroscience, University of Genoa, Genoa, Italy; ^6^Laboratory for Industrial and Applied Mathematics (LIAM), York University, Toronto, ON, Canada; ^7^Department of Mathematics and Statistics, York University, Toronto, ON, Canada

**Keywords:** Grit, physical education and sports, scale development, scale validation, students

## Abstract

**Background:**

Grit is a key concept in positive psychology and educational science. The construct measures two related constructs that are interest and effort. Several instruments have been developed to measure this construct in professional and educational contexts, but no tools have been developed considering specific contexts such as physical education and sport.

**Objectives:**

The objective of this study is to develop and test a measurement scale to assess Grit in the context of physical education and sport.

**Methods:**

Two exploratory (Phase 1) and confirmatory (Phase 2) samples were administered the 16-item PE-Grit scale in Arabic. In addition, the confirmatory sample also was administered the R-SPQ-2F two-factor learning approaches scale. The factor structure was examined first by exploratory factor analysis on the first sample and then by confirmatory factor analysis on the second sample. Reliability testing was performed by checking internal consistency simultaneously by the three indices: McDonald's ω, Cronbach's α and Gutmann's λ6. Concurrent validity was checked by Pearson's correlation between the PE-Grit and the two dimensions of the SPQ-2F.

**Results:**

After the exploratory factor analysis, which identified the factors and gave a preliminary validation of the designed instrument, confirmatory factor analysis was performed on three hierarchical models to be able to identify the best fitting model. A third-order hierarchical model with two physical and academic components each formed by interest and effort presented the best fit indices: chi X2 = 192.95 (*p* < 0.01), and the X2/DF = 1.36; GFI = 0.99; AGFI = 0.99; CFI and TLI close to 1; RMSEA = 0.025. In addition, McDonald's ω, internal consistency, and Gutmann's λ6 ranged from 0.78 to 0.86 for all four scale dimensions.

**Conclusion:**

The PE-Grit scale displays adequate factor structure, good reliability, and acceptable concurrent validity and can be administered to assess Grit in physical education and sport students.

## Introduction

Today, physical education, as a regular and institutionalized physical activity, has become central to the school and university system in most countries. Indeed, there is a growing interest in the role of physical education. Physical education is no longer focused solely on its impact on physical and mental health but has expanded to prepare young people for the demands and challenges of everyday life (1).

It has been argued elsewhere that physical education teaching is a multiple setting (2, 3), which differs greatly from classroom teaching in terms of characteristics, professional tasks, and specificity of the content offered in learning sessions (4). In detail, to teach physical education, a wide variety of practical and theoretical expertise must be considered (5). In fact, specific requirements must be considered in the student's training program (6). Preparing a future physical education teacher essentially requires multiple practical and theoretical knowledge to develop the necessary professional skills (7). Therefore, student training and learning in this field draws on several interdisciplinary (8) and transdisciplinary fields (9). As an example, the student must have theoretical knowledge in psychology, sociology, pedagogy, statistics, movement science, and biology, in addition to practical training in sport. Likewise, practical training requires mastery of multiple expertise in team and individual sports (10, 11). Therefore, a significant body of research has argued that physical performance and the development of various psychomotor, emotional, and cognitive skills are essential for success. In this regard, students in this field are exposed to adverse conditions similar to those of athletes: for example, physical fatigue (12), pain that is caused by many physical and athletic activities (13), and even high pressure on the lumbar spine (14, 15). Similarly, the student is also exposed to mental fatigue (16, 17), circadian rhythm disruption (18), sleep disturbance, and insomnia related to high activity levels (19).

Indeed, success in this field requires a great deal of courage and Grit. As a matter of fact, in college, schools, and universities, Grit has become a central concept in positive psychology (20), evaluated as a critical predictor of success, and for academic performance (21, 22).

As inspired by the initial research of Duckworth et al. (23) and Duckworth and Quinn (24), the concept of Grit, as an indicator and essential component of success and achievement, has been considered as one of the main concerns of personality investigations and educational psychology among college and undergraduate students along the last decade (24–26). Grit as a favorable individual trait predicting success and achievement has emerged in a variety of contexts and cultures (27). Grit has been used to identify achievement in domains typically assessed as difficult and requiring hard work (23, 24).

In the field of education, Grit encompasses the concepts of passion and long-term perseverance and is becoming an increasingly important characteristic in preparing students for academic or university success (28–30). It can also be defined as effort and determination to achieve goals (31). Grit is negatively associated with stress (32) and burnout (33). Indeed, it describes the ability to recognize good performance, despite all individual constraints, to achieve a planned goal. The most noted early work perceived Grit as a construct formed by two factors, consistency of interest and perseverance in effort (23, 24, 34).

Consistency of interest refers to the extent to which individuals maintain a preoccupation with achieving long-term goals, whereas the persistence of effort (PE) refers to the extent to which individuals can maintain their efforts to achieve these goals regardless of the challenges and failures they encounter (35). Although defined as “an individual's tendency to persistently pursue long-term goals despite challenges or obstacles” (36), Grit must be distinguished from the related concepts of resilience, or “ability to overcome,” “acute,” “chronic,” “dynamic,” hardships and “everyday resilience” (37). Grit as a stable personality trait has been contested by some researchers who have found that Grit varies over time (38), leading to a reconceptualization of the construct as an out-of-domain cognitive process that may be appropriate for intervention (29).

Overall, Grit has been designed in several measurement instruments in a hierarchical model with two correlated dimensions that are the persistence of effort (PE) and consistency of interest (CI) (39–42). The first refers to the extent to which individuals exert long-term efforts to challenge contextual barriers (24), whereas the second measure shows the tendency of a subject to adopt a long-term choice of interests (24).

Two measurement scales that were initially developed and widely used in different contexts are as follows: the original Grit (Grit-O; 12 items) and the short Grit scale (Grit-S; 8 items), both developed by Duckworth and colleagues (23, 24). Other instruments have been developed in the context of education, such as the Academic Grit Scale (29) and the Grit Scale for Children and Adults (41). However, Datu et al. (43) suggest other alternative measures of Grit that have three factors the Triarchic Model of Grit Scale (TMGS) by adding the factor adaptability to situations. Even though, Grit-O and Grit-S remain the most widely used measures in different contexts and countries (see Arco-Tirado et al., 2018). For example, in sport, according to the scoping review elaborated by Cormier et al. (44), previous research on the measurement of athletes Grit in different levels of practice was achieved by these instruments.

To measure the concept of Grit among PE students, it is necessary to focus mainly on the content of their educational background. Indeed, PE education differs from other education, and this may be mainly due to the physical component in the educational process. If we measure the persistence and interest of these students, this component should not be overlooked. Referring to previous reports, in a particular academic context, student motivation, passions, and interests have been associated with the provided learning content (45–48).

Physical education students are a specific group of individuals who were engaged in physical activity in two contexts: studies and assessment. Successful performance in physical and sporting activities is critical to the student's success in school.

It is worth adding that the practical sessions of physical education teacher training take place in specific structures outside the classroom (e.g., gymnasiums, running tracks, soccer fields, etc.) (49). Scholar sport facilities and their variances from country-to-country can create specific conditions for physical education and sometimes disadvantageous environments due to lack of comfort (e.g., poor countries). In addition, outside the classroom, the PE student may be exposed to unfavorable weather conditions (e.g., extreme heat or cold), whereas students in other disciplines benefit from sheltered classrooms.

In the academic field of physical education, the Academic Grit Scale cannot assess the concept since it does not include interest and physical effort. Indeed, interest and physical effort cannot be mistaken for academic interest and effort.

Therefore, the purpose of this study was to develop and evaluate a measurement scale to assess Grit in this field. In this procedure, which considered as preliminary in the context of physical education, the factorial structure, the internal consistency, and the concurrent validity will be considered. In the academic context, each student has his own learning strategy: deep learning strategies and surface learning strategies (50). Therefore, to show the concurrent validity of the PE-Grit, we proceed to examine the association of this scale with the R-SPQ-2F scale, which is a measure of learning strategies. We justify this choice by the associations that have been found recently in several studies between Grit and learning strategies (33, 51, 52).

## Materials and Methods

### Measurements

#### The Development of the PE-Grit Scale

The development of the PE-Grit scale was conducted in the three steps listed below.

The first one consists of a review of the literature concerning the two-factor Grit in the academic context, its measurement instruments, and the specific characteristics in physical education and sports. In detail, the items of the Grit-S, Grit-O, and AGS measurement scales were considered (23, 24, 29). However, since the training and the assessment in physical education have theoretical subjects and physical practice (53–55), it was necessary to generate a pool of items that took into account on this specificity. Indeed, students in this field may have an interest in physical practice, whereas they may not have an interest in academic training (or vice versa). Likewise, the student can exert an effort which differs between the practical and theoretical sessions.

This work resulted in the development of a 16-item questionnaire in Arabic, which allows for the measurement of Grit through four context-specific dimensions, each comprising four items: interest in physical activity, interest in academic training, effort in physical activity, and academic effort (see Appendices).

In a second step, a focus group was formed by three independent and bilingual (English and Arabic) researchers as experts (one expert in humanities, two experts in applied educational sciences in physical education and sport) and two other Arabic and English academics as linguistic experts. The work of the group was to review the Arabic version and to develop an English version of the PE-Grit scale (see Appendix Table 1). During the process, the committee checked for any inconsistencies in the items in the two versions and made corrections if necessary.

The third step is a pilot study to examine the relevance of the items in the Arabic version, their face validity and to ensure their understandability by an exploratory sample.

The tool is evaluated on a seven-point Likert scale ranging from strongly disagree to strongly agree.

#### Arabic Version of the Revised Two-Factor Study Process Questionnaire R-SPQ-2F

The Arabic version of the R-SPQ-2F by Munshi et al. (56) is an assessment tool for learning approaches. It consists of 20 items that assess deep (10 items) and surface (10 items) learning approaches.

The surface concept means the acquisition of knowledge only with extrinsic motivation (i.e., students experience learning as an external duty necessary to succeed with minimal effort). In contrast, deep learning involves the acquisition of knowledge and understanding of underlying principles and mechanisms, critical thinking, and thus intrinsic motivation.

Data were collected on a five-point Likert scale. The characteristics of the scale in the exploratory factor analysis were satisfactory. However, no deep structure analysis was performed. Similarly, the internal consistency of the scale was good. Cronbach's α coefficients were 0.93 and 0.90 for the two factors, surface approach and deep approach, respectively.

### Data Collection and Procedures

Data were collected online from a set of students enrolled in the four universities of physical education and sports in Tunisia (*n* = 652). Those recruited for the study were divided into two groups to conduct two exploratory and confirmatory studies.

a) Exploratory data were collected from 170 students aged 22.40 ± 1.65 years randomly selected from the data. The subjects were recruited from both sexes, women (*n* = 88; 51.8%) and men (*n* = 82; 48.2%), belonging to the students at the Higher Institute of Physical Education and Sport of Tunisia. They were either athletes playing in several clubs and in different disciplines (34.70%), or heavy athletes (30%), or subjects who had never practiced sports in sports clubs (35.30%) who were admitted to a specific entrance examination allowing them to enroll in the institution.
b) Confirmatory data were collected from a total of 482 students who were aged 19–26 years (M = 21.94, SD = 1.80). Students of both genders are divided into three grades and have different sports experience: current athletes (17.43%), heavy athletes (40.66%), and subjects who have never played sports in a club (41.91%). All students who received practical and theoretical educational contents at the university and classes during that period were not suspended due to COVID-19.

Table 1 summarizes the distribution of recruited students according to the three variables: sport activity, grade level, and gender.

**Table 1:** Distribution of participants by sport practice, study level, and gender.

**Group**		**Sport practice**			**Study levels**			**Gender**	
		**Abundant athletes**	**Non-sports subjects**	**Sports subjects**	**First**	**Second**	**Terminal**	**Female**	**Male**
Exploratory sample	*n*	51	60	59	49	58	53	88	82
	%	30	35.30	34.70	28,82%	34,12%	31,18%	51.8%	48.2
Confirmatory sample	*n*	196	202	84	195	174	113	272	210
	%	40.66	41.91	17.43	40.55	36.01	23.44	56.43	43.57

All data were collected during an online survey designed using Google Forms to protect students from COVID-19. The survey includes a demographic form in which age, gender, education level, and athletic experience were indicated. This process also has an advantage in that the responses can be retrieved on a Microsoft Excel page. The email addresses of the students were obtained by the use of the university system. In addition, no further information was provided (e.g., the student's first and last name).

### Statistical Analyses

Statistical analyses were performed using IBM SPSS version 26.0 for Windows. The reliability of the instrument was tested by the open-source software JASP. Whereas Lavaan's R package (R Studio) was adopted for confirmatory factor analysis, preliminary data analysis was performed by Skewness and Kurtosis normality tests. Exploratory sample responses were performed by unweighted least squares method with Promax rotation and Kaiser normalization. Instrument reliability was obtained by calculating the internal consistency coefficients: Cronbach's α coefficient, McDonald's ω coefficient, and Gutmann's λ6 coefficient.

The recommended threshold for these indexes is 0.70 for acceptability and 0.80 for good reliability. The structure of the confirmatory sample questionnaire was performed by confirmatory factor analysis (CFA), and diagonally weighted least squares (DWLS) was used in this study as an estimation technique (57). Several CFA indices were selected to examine the model: (1) the X2, (2) the X2/DF, (3) the goodness-of-fit index (GFI), (4) the goodness-of-fit index (AGFI), (5) the comparative fit index (CFI), (6) the Tucker–Lewis index (TLI), and (7) the root means square error of approximation (RMSEA).

The X2 should not be significant; however, this criterion is highly criticized on large samples, whereas the X2/DF is widely used and should be less than or equal to 2.

According to the recommendations of Hu and Bentler (58), GFI and AGFI must have values greater than 0.90 to accept the model. TLI and CFI values greater than 0.95 represent a good model fit. RMSEA should be <0.06 for a good model fit and <0.08 for an acceptable model fit (58, 59).

Concurrent validity was tested by examining the association between the four PE-Grit factors and the R-SPQ-2F scale. To examine these associations, we used low (<0.35), moderate (between 0.36 and 0.67), and strong (>0.67) thresholds for Pearson's correlation coefficients (60).

### Ethical Statement

This work has received approval from the Ethics Committee of the Research Unit, Sportive Performance, and Physical Rehabilitation, High Institute of Sports and Physical Education, Kef, University of Jendouba, Jendouba, Tunisia and received ethical clearance from the UNESCO Chair Health Anthropology Biosphere and Healing Systems, University of Genoa, Genoa (Italy), the Higher Institute of Sport and Physical Education of Kef, Kef (Tunisia), and the Higher Institute of Sport and Physical Education of Sfax, Sfax (Tunisia). The proposal has been also approved by the Jendouba University Ethics Committee and was undertaken following the legal standards of the Helsinki declaration in 1964 and its corresponding amendments.

## Results

Factor analysis was used to extract the four factors from the correlation matrix. All 16 items were subjected to factor analysis using extraction by the unweighted least square method, Kaiser normalization, and Promax rotation. Only the elements with loads equal to or greater than 0.50 were retained.

This analysis resulted in 4 factors with eigenvalues greater than 1, a Kaiser–Meyer–Olkin measure of sampling adequacy index, KMO of 0.88, Bartlett's sphericity test, and chi-square test of value 1,257.81 (ddl = 120; *p* < 0.001).

The factorial solution resulted in four factors that explained 69.15% of the total variance.

The first factor explained 35.51% of the total variance (eigenvalue = 5.68), whereas the explained variances were 16.94% (eigenvalue = 2.71), 7.37% (eigenvalue = 1.49), and 7.37% (eigenvalue = 1.18). Table 2 presents the descriptive statistics, the normality coefficients, and factor loadings (lambda) of the item scores obtained in the exploratory phase.

**Table 2:** Descriptive statistics, normality test, and factor loadings of exploratory factor analysis.

**Items**	**Mean**	**SD**	**Skewness**	**Kurtosis**	**Lambda**
PHI1	4,36	1,55	−0,20	−0,52	0,78
PHI2	4,44	1,58	−0,18	−0,63	0,75
PHI3	4,41	1,56	−0,14	−0,86	0,72
PHI4	4,41	1,45	−0,22	−0,50	0,72
ACI1	4,23	1,68	−0,12	−0,72	0,80
ACI2	4,34	1,69	−0,24	−0,77	0,80
ACI3	4,30	1,43	−0,20	−0,92	0,74
ACI4	4,26	1,74	−0,15	−0,83	0,79
PHE1	3,88	1,72	−0,05	−0,89	0,76
PHE2	3,99	1,69	−0,01	−1,02	0,73
PHE3	3,63	1,48	0,20	−0,55	0,73
PHE4	3,69	1,50	0,20	−0,63	0,78
ACE1	3,44	1,58	0,28	−0,62	0,78
ACE2	3,24	1,65	0,55	−0,50	0,75
ACE3	3,45	1,59	0,21	−0,78	0,78
ACE4	3,35	1,62	0,30	−0,75	0,81

*PI, Physical interest; ACI, academic interest; PPE, physical practice effort; ACE, academic effort. After examining KMO, factor loadings, and scree plot (Appendix Figure 1), the factorial solution was retuned without deleting any items*.

### Reliability

The McDonald's ω internal consistency indices for the four dimensions vary between 0.86 for the AC, effort dimension, and 0.83 for PH. interest one. This shows a good consistency of the 4 dimensions of the scale. Also, Cronbach's α values are good with a minimum value of 0.83 for the academic effort dimension (see Table 3).

**Table 3:** Internal consistency of PE-Grit scale.

**Estimate**	**McDonald's ω**	**Cronbach's α**	**Guttman's λ6**	**Average interitem correlation**	**Mean**	**SD**
PH.Interest	0,83	0,83	0,78	0,55	17,62	4,99
PH.Effort	0,86	0,86	0,82	0,61	17,13	5,50
AC.Interest	0,84	0,84	0,80	0,56	15,19	5,24
AC.Effort	0,86	0,86	0,82	0,61	13,48	5,41

For Gutmann's λ6 index, the scores vary from 0.78 to 0.82 for the fourth and the first dimensions, respectively (see Table 3).

### Confirmatory Factor Analysis

**Figure 1:**
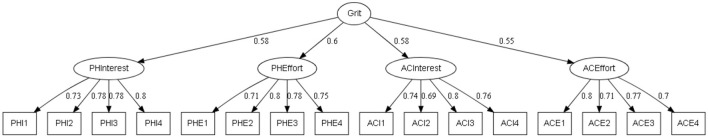
Second-order confirmatory factor analysis of the PE-Grit. All parameters are standardized and significant at the 0.01 level.

**Figure 2:**
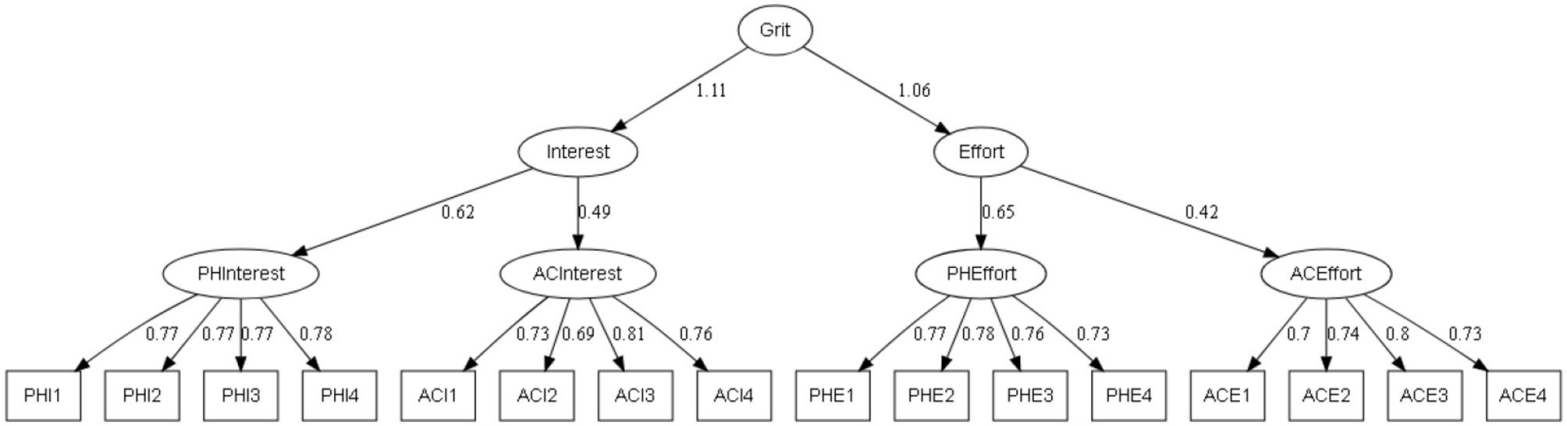
Third-order confirmatory factor analysis of the PE-Grit (M2). All parameters are standardized and significant at the 0.01 level.

**Figure 3:**
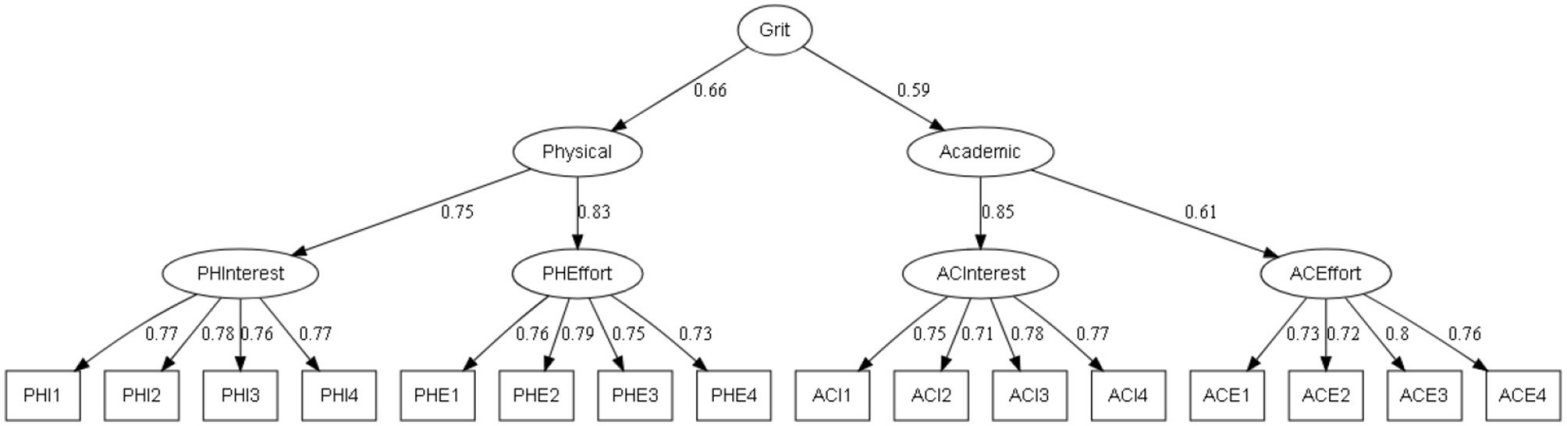
Third-order factor analysis of the PE-Grit (M3). All parameters are standardized and significant at the 0.01 level.

To validate the generalizable of the factor structure, confirmatory factor analysis was carried out on three different models: (a) a model with four factors which form a total score (M1, see Figure 1) (b) a third-order model (M2, see Figure 2) where the total Grit score is formed from two scores of interest (physical interest and academic interest) and effort (physical effort and academic effort) and the third model also of third-order (M3, see Figure 3) is formed from a physical dimension (physical interest and physical effort) and an academic dimension (academic interest and academic effort).

### Review of Adjustment Indices

The models shown in Figures 1–3 exhibit excellent factor loadings which are greater than 0.71 which are recommended by Comrey and Lee (61).

For model M1, the absolute goodness-of-fit indices X2/DF and RMSEA were not adequate. Indeed, the value of X2/DF was 3.82 > 2, and both error indices were greater than 0.60. Likewise, the TLI and CFI indices were slightly below the recommended threshold for both indices (>0.95). However, the GFI and AGFI indices were adequate. Following these results, the M1 model will be rejected and other models must be examined [see Hu and Bentler, (62)].

Referring to the recommended thresholds, the M2 model exhibits good fit indices with the exception of X2/DF and measurement errors.

Likewise, the M3 model provided the best-fit indices that are faithful to the recommended standards, but with a smaller chi X2 = 67.54 (*p* < 0.01), and the X2/DF = 0.68.

In addition, the indices of GFI = 0.994 and AGFI = 0.992. The parsimony-adjusted measures indices CFI = 0.99 and TLI = 0.99 were close to 1, which justifies the excellence of the model. The RMSEA and the RMR values were 0 and 0.033, respectively. These values indicated a good adjustment index.

### Concurrent Validity

For PH. interest, the results demonstrated significant associations (see Table 4). However, the correlation coefficient between this factor was low with deep (*r* = 0.19) and moderate with surface (*r* = 0.37) (see Table 4). Likewise, the results showed significant weak correlations between PH. effort with deep (*r* = 0.15) and PH. effort with surface (0.30) (see Tables 4, 5).

**Table 4:** Fit indices of the three models of PE-Grit scale.

**Model**	**X^**2**^ (p)**	**DF**	**X^**2**^/DF**	**GFI**	**AGFI**	**TLI**	**CFI**	**RMSEA**	**[Lo90-Hi 90]**
M1	393.89 (*p* < 0.01)	103	3,82	0.964	0.952	0.937	0.946	0.077	0.069–0.085
M2	334.31 (*p* < 0.01)	99	3,38	0.969	0.958	0.947	0.956	0.070	0.062–0.079
M3	67.54 (*p* < 0.01)	99	0,68	0.994	0.992	1	1	0.000	—

**Table 5:** Correlation between the dimensions of the PE-Grit scale and the two factors, deep approach, and surface approach.

**Dimension**	**PH.Interest**	**PH.Effort**	**AC.Interest**	**AC.Effort**	**Deep**
PH.Interest					
PH.Effort	0.50^**^				
AC.Interest	0.26^**^	0.19^**^			
AC.Effort	0.11^*^	0.23^**^	0.41^**^		
Deep	0.19^**^	0.15^**^	0.43^**^	0.31^**^	
Surface	0.37^**^	0.30^**^	0.08	0.03	0.29^**^

* *p < 0.05*;

** *p < 0.01*.

The results showed a significantly moderate and weak relationship between AC. interest with deep (*r* = 0.43) and AC. interest with surface (*r* = 0.08), respectively. Finally, AC. effort was associated with deep (*r* = 0.31), and no association was demonstrated between the PE-Grit factor with surface (see Table 5).

## Discussion

The purpose of this study was to develop and test a measurement scale to assess Grit in the context of physical education and sport. A 16-item, four-factor scale was developed and empirically tested to assess its psychometric properties in a near-target population of physical education and sport university students.

A first second-order model was tested by confirmatory factor analysis and proved to be reliable. Subsequently, two third-order hierarchical models were evaluated to examine the most appropriate structure to represent the empirical data. A third-order model with two physical and academic components better displayed the data.

Afterward, the internal consistency was assessed to test the reliability of the instrument. The results proved that the four dimensions of the instrument have adequate internal consistency. Finally, concurrent validity was assessed using the Pearson's correlation matrix between the four PE-Grit dimensions and the two factors of the R-SPQ-2F scale.

Pearson's correlation results showed that physical interest has a slightly positive correlation with the deep approach and a moderate correlation with the surface approach. In addition, academic interest (AC. I) is moderately associated with the deep approach, whereas a weak association of physical effort with both factors of the R-SPQ-2F was proven. Similarly, a moderate correlation between academic interest (AC. E), academic effort, and deep approach was demonstrated. Finally, no correlation between academic effort (AC. E) and superficial approach was found. These results confirm that students who are more interested in sport activities have a surface approach and therefore extrinsic motivation. Additionally, students who are more motivated to learn have a more academic interest in their academic training.

In line with the latter findings, Datu et al. (43) postulate that many efforts in the university context require students' persistence to achieve long-term goals. They emphasize the importance of being passionate and having the determination to achieve their goals through sustained personal effort in school or university (63). In addition, Tang et al. (64) research showed that Grit is associated with high academic commitment and achievement.

In contrast, King and Ganotice (65) in their study of Filipino students concluded that the deep approach did not necessarily influence academic performance more than the surface approach. Indeed, in this Asian culture, students put a lot of effort into preserving their face and avoiding poor performance that is frowned upon. In this cultural context, academic Grit can be associated with encouragement and respect for the effort exerted to keep face.

Likewise, in line with initial validations of the Grit-O (23) and Grit-S (24) scales, the PE-Grit measure exhibits a multidimensional structure. Similarly, the results of exploratory factor analysis on the Grit Scale for Children and Adults (GSCA) supported the multidimensionality of the instrument (41). In agreement with these studies suggesting that the two factors, interest and effort, are correlated, our first-order model did not show good fit indices. However, the psychometric review of the Academic Grit Scale conducted by both exploratory and confirmatory factor analyses supported a unidimensional structure (29). In addition, factorial reviews of the Triarchic Model of Grit Scale (TMGS) supported a first-order model (43).

Actually, most of the studies across time and countries have focused on the Grit-S. In fact, the tool has provided a good internal consistency, adequate test–retest stability, and good other psychometric proprieties [for example, (36, 40, 66)]. In addition, Grit-S total score was associated with educational level. Despite this, too many controversies of the structure were reported. For the sake of clarity, Gonzalez et al. (67) concluded through parallel analysis, a measure of instability, extrinsic convergent validity, and item response theory models (on two US samples) that the short version of the Grit is unidimensional. In another study, the theory item analysis of a Russian version (68) revealed two uncorrelated dimensions. Much more, in a German version evaluated among students, Schmidt et al. (36) proved a second-order structure by confirmatory factor analysis. It also found concordances of the scale scores with self-efficacy and general academic self-concept.

Furthermore, in another study by Clark and Malecki (29) on a sample of adolescents, an Academic Grit Scale was subjected to empirical examination. Results from exploratory and confirmatory factor analyses supported second-order structure. Internal consistency was high and positive correlations between academic Grit and academic achievement were reported.

The concept of Grit has always been linked to academic performance (69). To that end, Duckworth and Gross (70), Hochanadel and Finamore (71), and Keegan (72) encourage parents and teachers to teach Grit to children and students. Thus, interventions to improve it are always welcome. However, other researchers argue that other aspects of learning such as learning conditions, mentor's self-efficacy, and access to resources and thus the intervention in this regard will be more desirable (73, 74).

In addition, the study by Steinmayr et al. (75) showed that the effect of Grit on achievement was weaker than that of self-perceived ability and academic commitment. Similarly, an international study in several populations showed that sociodemographic factors, health behaviors, and psychology were associated with academic performance (22, 76).

In conclusion, all of the instruments were tested and validated with a common one-factor and mostly two-factor designs, and high scores on these scales were positively associated with academic or school performance. However, in contexts that also require physical performance, a mismatch may arise between academic and physical interest, similarly between academic effort and physical effort. As a matter of fact, students in physical education and sports in Tunisia have several subjects based on physical practice that can increase their grades and meet the conditions for success. Most of these students have integrated the academic course through a sporting career. Therefore, for them, they can succeed thanks to the grades achieved by the practical disciplines.

Several perspectives and variables need to be examined in the context of physical education and sport such as gender, sport experience, and grade repetition. Indeed, in the context of university medical students, a study by Alzerwi (77) showed that students' Grit scores were higher than those of men and also differences related to repetition were highlighted.

We are aware that our research has some limitations. The first is the examination of the relationship between PE-Grit with different versions of Grit which was not conducted. Second, the multigroup scale sensitivity was not conducted to see the differences between dropout athletes and student athletes. Third limitation of our study is that the version presented in the English manuscript was not empirically tested, and only the Arabic version was tested. The other limitation of the study was the factor variability between the variables gender, sports background, and education level which was not achieved. Finally, it is preferable in a prospective study to examine the association between personality traits and PE-Grit.

### Implications and Future Directions

This study has raised the challenge of developing a scale to measure Grit in the specific context of physical education and sports for future school teachers. The integration of the physical component with two factors, interest and effort, allows for a better assessment of Grit in this field. Future studies on the concept of Grit in this academic context may consider the intensity and frequency of physical exercise in the teaching of future physical education teachers. Indeed, the physical load that exerted during learning sessions may influence both interest and effort factors of the physical dimension and consequently on the total Grit score. It is interesting to conduct studies in this direction to understand how the student plans his or her goals.

Finally, the motivation to exercise, the culture for exercising, the enjoyment of exercising, the credits attributed to physical activities, and the grading systems (e.g., the coefficients of the subjects taught) may be different from one country to another. It is therefore worthwhile to understand the concept in different countries and cultures.

## Conclusion

The examination of the beneficial role of positive psychology instruments such as the Grit in specific academic contexts is necessary, as they provide insight into learner performance. The PE-Grit scale developed, obeys an adequate factor structure, good reliability, and acceptable concurrent validity and can be administered to assess Grit in physical education and sport students.

## Data Availability Statement

The original contributions presented in the study are included in the article/supplementary material, further inquiries can be directed to the corresponding author/s.

## Ethics Statement

This work has received approval from the Ethics Committee of the Research Unit, Sportive Performance, and Physical Rehabilitation, High Institute of Sports and Physical Education, Kef, University of Jendouba, Jendouba, Tunisia and received ethical clearance from the UNESCO Chair Health Anthropology Biosphere and Healing Systems, University of Genoa, Genoa (Italy), the Higher Institute of Sport and Physical Education of Kef, Kef (Tunisia), and the Higher Institute of Sport and Physical Education of Sfax, Sfax (Tunisia). The proposal has been also approved by the Jendouba University Ethics Committee and was undertaken following the legal standards of the Helsinki declaration in 1964 and its corresponding amendments. The patients/participants provided their written informed consent to participate in this study.

## Author Contributions

NG and NB conceived the experiment. NG, NC, AT, and NB collected and analyzed data. All authors critically revised the manuscript. All authors contributed to the article and approved the submitted version.

## Conflict of Interest

The authors declare that the research was conducted in the absence of any commercial or financial relationships that could be construed as a potential conflict of interest.

## Publisher's Note

All claims expressed in this article are solely those of the authors and do not necessarily represent those of their affiliated organizations, or those of the publisher, the editors and the reviewers. Any product that may be evaluated in this article, or claim that may be made by its manufacturer, is not guaranteed or endorsed by the publisher.

## Figures and Tables

**Figure A1:**
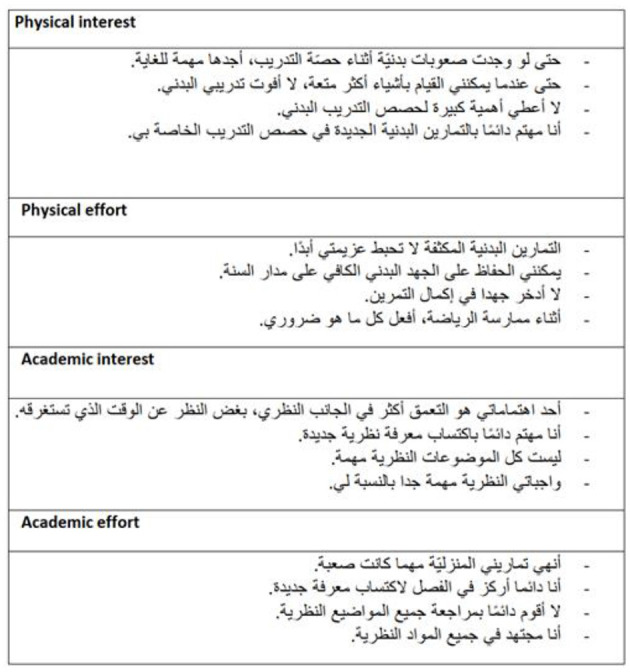
The Arabic version of the PE-Grit.

## Appendices

**Table A1:** English version of the PE-Grit.

**Physical interest**
- Even if I find physical difficulties during the training session, I find them very important. - Even when I can do more fun things, I do not miss my physical training. - ^*^I do not give much importance to physical training sessions. - I am always interested in new physical exercises in my training sessions.
**Physical effort**
-Intense physical exercise never discourages me. - I can maintain adequate physical effort all year round. - ^*^I spare no effort in completing the exercise. - During the physical practice, I do whatever is necessary.
Academic interest
- One of my interests is to go deeper into the theoretical side, regardless of the time it takes. - I am always interested in acquiring new theoretical knowledge. - ^*^Not all theoretical subjects are important. - My theoretical duties are very important to me.
**Academic effort**
- I finish my home exercises, no matter how hard they are. - I always focus on class to acquire new knowledge. - ^*^I do not always revise all theoretical subjects. - I am diligent in all theoretical subjects.

* *The item score must be reversed*.
